# Humorous Coping With Unrequited Love: Is Perspective Change Important?

**DOI:** 10.3389/fpsyg.2021.653900

**Published:** 2021-06-25

**Authors:** Werner Greve, Johanna Hauser, Farina Rühs

**Affiliations:** Institute for Psychology, University of Hildesheim, Hildesheim, Germany

**Keywords:** cognitive means of coping, alleviative effect of humor, flexible goal adjustment, unrequited love, self-esteem

## Abstract

A large number of studies suggest that humor is associated with mental well-being and effective as a means of coping. However, it is less well-understood which mechanisms are effective for this particular function of humor. The present study examines whether processes of change of perspective, which are often regarded as constitutive for humor, could be an effective coping-factor when facing unrequited love as a specific psychological burden. In a questionnaire study, *N* = 148 persons aged 18–65 years (*w* = 96) with actual or past experiences of unrequited love reported on their subjective burden due to this experience, their self-esteem and satisfaction with life, two scales for humor (Multidimensional Sense of Humor Scale: MSHS, and a self-constructed scale: Humorous Change of Perspective, HCOP) and a coping scale which measure change of perspective in the confrontation with goal blockages (Flexible Goal Adjustment, FGA). Results indicated that the burden of unrequited love [operationalized objectively as actuality of experience (dichotomous) or subjectively as burden experienced] and both indicators of well-being were negatively associated. Multiple regression analyses showed that humor was a significant moderator of this relationship in nearly all combinations of operationalizations of humor and indicators of well-being: Higher levels of humor are associated with better well-being even when the perceived burden was high. In addition, the study examined whether the coping effect of humor can be partly or mainly attributed to the individual's capacity to perspective change as captured by FGA. When including this scale as a covariate in the regression models, the moderation effect for MSHS did not persist; however, for HCOP the moderation effect remained unchanged: the moderator effect of humorous change of perspective proved to be independent of FGA. Taken together the results suggest that perspective-changing skills play a significant role in the coping effect of humor in dealing with psychological burdens. However, depending on which humor facet is measured, the entailed perspective change may or may not appear to go beyond what the individual's FGA can account for. This suggests that the coping effect caused by humorous change of perspective includes aspects that are also discussed for other coping resources as well as its own, humor-specific aspects. Potential avenues for future studies are discussed both with respect to the necessity for replication and extension of the present study and to the determination of other potential alleviativing effects of other facets of humor.

## Introduction

### Humor as a Resource

The assumption that humor is not only enjoyable for all who experience it, but is also a useful resource for those who have it, is as old as human laughter (Ruch, 2008; Hurley et al., 2011; Martin and Ford, 2018). The finding that humor can be helpful when coping with stress, burdens, threats, and other challenges is more recent but widely documented. One does not have to agree with the traditional psychoanalytic assumption that humor is the most mature and most elegant defense mechanism (Vaillant, 1993) to recognize the functionality of humor in burdensome situations. A large body of research indicates a positive relationship between humor and psychological well-being (Martin and Lefcourt, 1983; Lefcourt and Martin, 1986; Nezu et al., 1988; Kuiper et al., 1993; Thorson et al., 1997; Cann et al., 1999; Ruch et al., 2010; Svebak, 2010; Cann and Collette, 2014; Samson et al., 2014; Fritz et al., 2017; for an overview, e.g., Ruch, 2008; Kuiper, 2012; Martin and Ford, 2018). Accordingly, a strong sense of humor is associated with, for example, a more positive self-concept and self-esteem as well as higher life satisfaction (Kuiper et al., 2004; Ruch et al., 2010, 2018b; Ozyesil, 2012). There is even evidence that humor supports physical health (Svebak et al., 2006; Martin, 2008). Accordingly, Kuiper (2012) considers humor as a facet of individual resilience.

What is less clear, however, is how humorous coping with burdens can be explained. While the precise study of the processes at work here requires experimental designs, a helpful intermediate step may be to identify components of humor that make a difference in coping with burdens or threats. This requires a sufficiently sophisticated theory about the components of humor (possibly components of different varieties of humor); however, such a consensual theory of humor remains to be defined (Hurley et al., 2011; Martin and Ford, 2018), even though the debate about it predates psychology. Accordingly, the findings on the alleviative effect of humor are generally convergent, but in detail more heterogeneous than is desirable for a theoretical clarification of the factors that are effective here.

Several difficulties contribute to this. First, humor is regarded as a complex, multidimensional construct that includes emotional, cognitive, behavioral, and social components (e.g., Svebak, 1996; Thorson et al., 1997; Ruch, 2008; Martin and Ford, 2018). Accordingly, there are a variety of forms or styles, uses or intentions, and functions or effects of humor are distinguished, each differentially related to a broad variety of concepts, including personality traits, emotions, or social effects (Martin and Ford, 2018; Ruch et al., 2018a; Heintz and Ruch, 2019; for an overview, e.g., Ruch, 2008). An example of this is the distinction between “benevolent” and “malevolent” forms, or uses of humor (e.g., Ruch and Heintz, 2016). Positively connoted uses (forms) of humor that also dominate everyday understandings of humor as a positive skill (Craik et al., 1996; Perchtold et al., 2019) are typically associated with the aforementioned alleviative effects of humor are the. This conceptual heterogeneity is further reinforced by a variety of assessment methods that vary not only in their specific operationalization, but also with respect to the facets or functions of humor addressed (for instance, Ruch et al., 1996, 2018a; Martin et al., 2003; Kuiper et al., 2004; Heintz et al., 2018; for an overview, Martin and Ford, 2018).

In contrast, the question of which processes produce the alleviative effect of humor is less frequently studied. Martin (2007, 2008) identifies three main mechanisms underlying the positive influence of humor on well-being: physiological effects caused by the activity of laughter, the experience of positive emotions such as joy or exhilaration, and cognitive aspects such as the change in perspective (see also Martin and Ford, 2018). A number of papers have argued that in the context of coping with burdensome experiences, in particular cognitive aspects of humor are particularly important (e.g., Svebak et al., 2006; Svebak, 2010). According to this line of reasoning, humor leads to the shift of cognitive attention in favor of positive cognitions and associated positive emotions (Kuiper et al., 1993; Martin, 2007; Szabo, 2007; Strick et al., 2009; for an overview Ruch, 2008; Martin and Ford, 2018). Individuals with a more highly developed sense of humor rated their problems as less stressful and experienced fewer negative emotions (e.g., anxiety) than did individuals with lowly developed sense of humor scores, indicating that humor facilitates a different (i.e., relieving) perspective on problems. This assumption agrees with the suggestion that the ability and willingness for a positive appraisal of the initially threatening or stressful situation (Lazarus and Folkman, 1984) is significant for the coping effect of humor. Several studies have provided evidence that positive (re-)appraisal occurs more frequently in individuals with a more pronounced sense of humor and that it is functional for coping with burdens (Kuiper et al., 1995; Abel, 2002; Samson et al., 2014; Perchtold et al., 2019). For positive (re)appraisal, in turn, the cognitive ability to change perspective (e.g., reframing) is a necessary condition (Samson et al., 2014). Although not all humor theories agree that perspective change is a criterion (i.e., a necessary condition) for humor, a likely consensus is that it is a prototypical aspect of humor, perhaps just not just of all forms of humor (Martin, 2008; Hurley et al., 2011; Carroll, 2014; Martin and Ford, 2018).

At the same time, there is a large body of work showing that perspective change or reframing are particularly helpful for dealing with burdensome or threatening constellations when the underlying problem is a goal conflict or goal blockade that cannot be actively resolved (Brandtstädter and Rothermund, 2002; Brandtstädter, 2006; Heckhausen et al., 2010; Wrosch and Scheier, 2020). If it should be a valid consideration that the alleviative effect of humor is based, at least in part, on the fact that humor is associated with the individual capability for perspective change, then it should be possible to show that the alleviative effect of humor wanes in relation to a burdensome goal blockade if this aspect is out-partialized by a non-humor coping resource focusing on this aspect. Testing this consideration is the subject of the present study.

### Flexibility of Goal Adjustment as a Perspective-Changing Coping Resource

The two-process model of developmental regulation (Brandtstädter and Rothermund, 2002; Brandtstädter, 2006) distinguishes two modes of regulation by which the individual can reduce or eliminate problems (defined as discrepancy between an experienced actual state and a desired target state). In the assimilative response mode, the individual attempts to resolve a problematic situation through active, deliberate, and controlled problem solving. In doing so, personal goals and intentions are maintained and persistently pursued. In contrast, in the accommodative regulation mode, the reduction of the actual-target discrepancy occurs through “flexible goal adjustment” (Brandtstädter and Renner, 1990) to the given situation and the available options for action. Examples of this regulatory mode are devaluing the significance of the previous goal and upgrading alternative goals, changing one's own level of aspiration, or reinterpreting the problem situation on the basis of relieving cognitions. In short, an accommodative reaction requires a change of one's own perspective of the problem. It is required at the latest when assimilative efforts fail, problems cannot be overcome by active problem solving, or this would involve too much difficulty or cost. A large body of work has shown that the individual capability and tendency for accommodative regulation helps to reduce strain across different kinds of threats and burdens and across different age groups (e.g., Thomsen et al., 2015; Greve et al., 2017; Rühs et al., 2017; for review, Brandtstädter and Rothermund, 2002). Although this has not been a central focus of research, some results suggest that humor might be associated with accommodative coping. Thomsen (2016) reports a positive relationship between individual readiness to accommodate and coping humor (Martin and Lefcourt, 1983) in adolescents; the finding that this relationship becomes narrower with increasing age indicates that it is precisely the cognitively demanding aspects of humor that carry this relationship at least in part. Leist and Müller (2013) found an association between the self-enhancing and social humor style and flexible goal adaptation processes.

### Unrequited Love as a Goal-Blocking Burden

As social beings, humans possess a fundamental need to belong (Baumeister and Leary, 1995), in particular, the fulfillment of partnership happiness and positive partnership quality are considered central factors and predictors of psychological stability, life satisfaction, and well-being (Felser, 2007). How painful it is when this desire and need is not satisfied or is even actively denied by other people is something most people experience first-hand at some time in their lives. Unrequited love is an example of social rejection, which can arise in very different situations (e.g., crush on someone who is unavailable, break-up of existing relationship; see also Baumeister and Wotman, 1992; Baumeister et al., 1993, and Bringle et al., 2013 for a more in-depth description of such situations). Psychological research in recent years has shown that the experience of social rejection is generally associated with diverse negative consequences for the affected person in terms of psychological well-being (e.g., negative affect and reduced self-esteem; for meta-analyses/reviews see e.g., Blackhart et al., 2009, Gerber and Wheeler, 2009, and Leary, 2015; for an overview of the state of research on interpersonal exclusion and rejection Williams and Nida, 2017). Moreover, neuroimaging studies show that social rejection not only “hurts” in a figurative sense: The emotional pain associated with rejection parallels physical pain not only in the psychological burden it causes, but they also overlap in their somatosensory representation in the brain (Kross et al., 2011, but also see Eisenberger, 2015, for a summary of controversies regarding these finding). Whilst empirical research on effects of social exclusion from groups is very broad, studies which focus on the experiences of unrequited love are rarer. Nevertheless, the studies that do exist seem to confirm the general finding of negative psychological consequences also for this particular case of social rejection. One question that this field of research addresses in particular concerns the connection between romantic rejection/unrequited love and the rejected person's self-esteem. In a large-scale longitudinal study of adolescents and young adults over 3 years, Luciano and Orth (2017) found that the onset of a romantic relationship increased one's self-esteem substantially (if the relationship lasted at least a year), whereas a breakup reduced self-esteem for about a year. This fits with the findings of a recent study by Charlot et al. (2020), which showed that experience of repeated romantic rejection was associated with lower perceived mate value, among other factors. Other studies show that self-esteem not only matters as a potential consequence of romantic rejection experiences, but also has (conceptualized as a trait) a moderating role regarding the relationship of rejection and its consequences (see e.g., Waller and MacDonald, 2010; Park et al., 2011).

### Hypotheses

According to the line of argument presented so far, the present study combines three aspects. First, a broad assessment of humor is applied in order to not be bound to a specific humor theory and operationalization and to be able to assess as many different facets of humor as possible with regard to possible coping effects. Second, these effects will be examined in relation to unrequited love as a critical experience which is both plausible as burdensome and prototypical for a blocked goal. Since this burden has thus far only rarely been studied in relation to humor, this part of the present study is a replication of the available results for coping effects of humor. Third, it will be examined whether this (assumed) coping effect of humor in relation to this goal blockage can be shown to rely (at least partly) on change of perspective. This will be addressed by exploring the coping effect of humor whilst a coping resource that is precisely relevant for goal blockages and that is essentially based on a change of perspective is controlled for.

If unrequited love is regarded as a prototypical case of a burden caused by a goal blockage, if a central mechanism of coping with goal blockades, is to shift or change perspectives on the underlying problem (i.e., accommodative coping), and if humorous coping is essentially characterized by leading to or supporting a change of perspective, then the hypotheses are plausible that (1) humor buffers the burdens of an unrequited love, and (2) that this alleviative effect of humor can be attributed to the perspective changing-component of humor. To test these hypotheses, the first step is to replicate the relieving effect of humor (broadly assessed) in relation to unrequited love. As the second step, it has to be tested whether this effect is maintained after adding a specific coping resource that is essentially based on perspective change but not directly humorous (FGA) to the analysis. If the coping effect of humor were to disappear after the introduction of FGA (i.e., partialing out its effect), that is, if humor did not show any additional relief effect beyond this coping resource, this would indicate that this aspect (partially) explains the effect of humor. If, on the other hand, the alleviative effect is preserved, the alleviative effect of humor might not be based entirely on this aspect. In other words: this result would indicate an “incremental validity” (for a similar approach see Ruch and Heintz, 2013; Samson et al., 2014) of humor as a particular coping ressource beyond perspective change.

## Materials and Methods

### Study Design and Participants

In an observational study, participants filled in a cross-sectional, internet-based survey, which was generated by SoSci Survey (Leiner, 2020; Version 3.2.16) and was made available to participants from 6/8/2020 to 8/20/2020 via www.soscisurvey.de. Participants were recruited online via various social networks (e.g., Facebook, Xing) if they met following inclusion criteria: age between 18 and 65 years and (current or past) experience of unrequited love. The design of the study and the questionnaire obtained were approved by the ethical committee of the faculty of educational and social sciences of the University of Hildesheim (Proposal No. 134; letter of approval 4/20/2020). Data collection resulted in *N* = 148 completed questionnaires. The sample was relatively heterogeneous in terms of participants' age, ranging from 18 to 65 years (18–29 years: *n* = 72; 30–39 years: *n* = 52; 40–49 years: *n* = 10; 50–65 years: *n* = 14). Asked for their gender, *n* = 97 (65.5%) participants described themselves as female (34.5% male). The educational level of the participants was very high. Most participants (*n* = 139) reported that they graduated from secondary school and passed their “Abitur” (or had an equivalent qualification for university entrance) or even already had other higher education qualifications. Regarding the experience of unrequited love, 44 participants (29.7%) referred in their statements to a current experience and 104 participants (70.3%) to a past experience in their statements.

### Measures

The online questionnaire consisted of three parts. First, participants were asked for demographic data (such as gender, age, and educational background). They then provided information about various aspects of their experience of unrequited love (such as the time-scale of the experience or the severity of the perceived burden; some other aspects were obtained for more exploratory research but are not reported on in this study). In the third part, current self-esteem and life satisfaction (as indicators of subjective well-being) as well as humor (three different operationalizations^1^) and accommodative flexibility (as dispositional coping resource) were assessed using well-established scales (with one exception; see Humorous Change of Perspective).

#### Aspects of the Experience of Unrequited Love

##### Time-Scale of Experience

Participants were asked whether they were currently unhappily in love. If they answered yes, they were assigned a value of one for actuality. If they answered no but stated in the next question that they had experienced unrequited love in the past, they were assigned a value of zero.

##### The Burden of the Experience of Unrequited Love

For nine self-developed statements describing potential burdensome experiences during an episode of unrequited love (sample items: “My thoughts circled constantly around the beloved person.” and “I was often sad and lonely because of my unhappy infatuation.”) the participants indicated how much the statements applied to them (answers on a five-point scale from 1 = “does not apply at all” to 5 = “fully applies”). For the group of participants referring to a past experience, the items were formulated in the past tense, but otherwise the wording was not changed. A mean value was calculated for each participant to create a scale representing the subjective burden caused by their unrequited love experienced. The scale showed a good internal consistency (Cronbach's α = 0.86).

#### Humor

##### Sense of Humor

The Multidimensional Sense of Humor Scale (MSHS; Thorson and Powell, 1993; see also Martin and Ford, 2018) originally comprises 24 statements regarding different facets of the sense of humor in four broad dimensions: (1) humor production and social uses (example item: “I use humor to entertain my friends”), (2) humor as a coping strategy (example item: “Humor helps me cope”), (3) attitudes toward humorous people (example item: “people who tell jokes are a pain in the neck”), and (4) attitude toward humor itself (example item: “I like a good joke”). Participants indicate how much each statement applies to them (answers on a five-point scale from 1 = “does not apply at all” to 5 = “fully applies”). For this study, the scale was translated from English into German by one of the authors and back-translated by a native speaker to check for validity. In order to avoid a trivialization of the result (humor as a moderating coping resource), seven items of the original scale that directly address humor as a coping strategy were omitted. Considering the polarity of the items, a mean value of the remaining 17 items was calculated for each participant. The scale showed excellent internal consistencies (Cronbach's α = 0.92). As several studies in the past found different factor structures of the scale in varying samples (most studies showed one stable general factor and up to three other more specialized factors, e.g., José et al., 2007; Sousa et al., 2018), we performed an exploratory factor analysis. Results (for details see Supplementary Table 1) suggested that, in addition to the overall score, two subscales could be formed which correspond to the first dimension mentioned above (humor production and social uses, factor 1, 11 items) and a summary of the third and fourth dimension (attitudes toward humor and humorous people, factor 2, 6 items). The reason for the unidimensional (“overall”) usage of the MSHS in the present study was a theoretical one: The aim was to use an indicator that is as broad and heterogeneous as possible and that has a long tradition was used in order to replicate a coping effect that is as unspecific as possible. The very high internal consistency of the overall scale supports this decision. In addition, the heterogeneity of the “remaining” MSHS makes the replication of the buffer effect of humor more difficult and, hence, has a conservative effect with respect to the replication.

##### Humorous Change of Perspective

The central idea of the study was to identify the specific change of perspective assumed in humor as a cognitive component relevant for coping by controlling (and thus partialing out) this aspect by a coping scale that substantially (perhaps not only) addresses this aspect. Therefore, it was particularly important to use a humor scale (and to test its coping effect in relation to the burden studied here) that broadly encompasses diverse facets of humor, but, at the same time, does not specifically capture the coping effects of humor (which is why, as described above, these items were excluded from the MSHS). If the hypotheses about the study were true, controlling for FGA should reduce any possible coping effect of items specifically focusing on perspective-taking. We therefore decided to develop a short scale with exactly and only this focus. There are a number of (sub)scales that capture the coping effect of humor (including several scales that include items on perspective change); however, since the goal of the study was not to examine which coping humor scales overlap, in whole or in part, with the perspective change discussed in other coping scales in general and in FGA in particular, it seemed more consistent to use a specific short scale to control for this argument. Thus, a short scale consisting of four items (“it is easy for me to take a humorous perspective”; “a humorous perspective gives me easiness in life”; “I can adopt a humorous perspective in most situations”; “I quickly succeed in adopting a humorous perspective”) was constructed by the authors. Participants indicated how much each statement applies to them (answers on a five-point scale from 1 = “does not apply at all” to 5 = “fully applies”). An exploratory factor analysis indicated that the four items all cluster on one factor (for details see Supplementary Table 2). Thus, a mean value was calculated for each participant. The resulting scale showed an excellent internal consistency (Cronbach's α = 0.90).

#### Accommodative Flexibility

To assess the accommodative flexibility as a dispositional coping resource, Brandtstädter and Renner's (1990) flexible goal adjustment (FGA) scale was used. The scale consists of 15 items that contain self-statements regarding how to deal with situations in which one's own goals or wishes are blocked or can no longer be implemented as planned (example item: “Even during great distress, I often still find a meaning in life”). Participants indicated how much each statement applies to them (answers on a five-point scale from 1 = “does not apply at all” to 5 = “fully applies”). Considering the polarity of the items, a mean value was calculated for each participant. In the present study the internal consistency of the scale was excellent (Cronbach's α = 0.91).

#### Self-Esteem

The German version of the Rosenberg Self-Esteem Scale (RSES; Rosenberg, 1965; German version by Ferring and Filipp, 1996) was used to measure the participants' global self-esteem. It consists of 10 self-evaluating-statements (example item: “I possess a number of good qualities”). For each statement, the participants indicated how much it applies to them (from 1 = “does not apply at all” to 4 = “fully applies”). Considering the polarity of the items, a mean value was calculated for each participant. The scale showed an excellent internal consistency (Cronbach's α = 0.93).

#### Life-Satisfaction

The German version of the Satisfaction with Life Scale (SWLS; Diener et al., 1985; German version by Janke and Glöckner-Rist, 2014) was used to measure life satisfaction in the context of the theory of subjective well-being (Diener, 1984). According to this theory satisfaction with life is a multifactorial construct with affective and cognitive components. The scale consists of five statements (example item: “I am satisfied with my life.”), for each of which the participants indicate on a seven-point scale to what extent it applies to them (1 = “does not apply at all,” 7 = “applies completely”). To create a scale all values were summed up for each participant. In the present study the internal consistency of the scale was excellent (Cronbach's α = 0.91).

### Analytic Strategy

All analyses were performed using JASP (JASP Team, 2020). In a first step descriptive statistics of main study variables, group comparisons (participants that experienced unrequited love in the past vs. participants that are currently unhappy in love) and bivariate correlations were calculated to check whether there were any abnormalities in the distribution and expression of the variables that would have to be considered in the following hypothesis tests.

To test the first hypothesis that humor moderates the relationship between the burden of unrequited love and well-being (such that higher levels of humor are associated with a weaker negative relationship between indicators of burden and well-being), multiple regression analyses were performed. Since the study applied two different measures of humor (MSHS and HCOP), two different indicators of burden (actuality of experience and subjective burden due to unrequited love), and two different indicators of well-being (self-esteem and satisfaction with life), a total of eight different regression analyses were calculated as follows: The respective indicator of well-being was regressed on one indicator of burden, one indicator of humor and their interaction at one time (all continuous variables forming interaction terms were mean-centered before they were used in the analyses). This procedure was performed in the same way for all indicator combinations. If the moderator hypothesis is correct, the interaction-term of burden^*^humor should be a statistically significant predictor of the indicator of well-being used in that model. As recommended by Aiken and West (1991), statistically significant interactions were probed using simple slope analyses to further examine the directionality of the moderation. To do so, additional slopes for values of the used indicator of humor one SD below and above the sample's mean were calculated and tested against zero. All results were considered to be statistically significant when *p* < 0.05.

To address the second hypothesis regarding the role of perspective change for humor functioning as a coping-resource, the eight regression models for testing the first hypothesis were extended as hierarchical regression models by a step in which FGA was added as a covariate in the model. If the moderation effect is driven primarily by aspects of humor similar to those captured in the measure of the ability to flexibly change perspectives, the addition of this variable as a covariate should result in the interaction term no longer being a statistically significant predictor of the respective indicator of well-being. If the interaction term remains a statistically significant predictor in this model, it would suggest that also other (possibly more specific) aspects of humor are relevant to the coping effect that are not captured via the FGA measure. Again, statistically significant interactions were followed by simple slope analyses following the pattern already described for testing the first hypothesis. All results were considered to be statistically significant when *p* < 0.05.

## Results

### Descriptive Statistics, Bivariate Correlations, and Group Comparisons

Means, standard deviations, minimum, and maximum values of relevant study variables as well as bivariate correlations are shown in Table 1. Because application of the Shapiro-Wilk test revealed that all study variables were not normally distributed (all *p* < 0.002, a visual inspection as well as the calculation of skewness showed that most variables were distributed more or less to the right, −0.788 < Skewness < −0.450), Spearman's rank correlation coefficient was chosen to measure correlation. Both measures indicating burden of unrequited love (actuality of experience and subjective burden due to unrequited love) showed weak to moderate negative associations with the two indicators of subjective well-being (self-esteem and satisfaction with life, *r*_s_ range from −0.239 to −0.359, all *p* < 0.01). Both measures of humor where moderately associated with each other (*r*_s_ = 0.666, *p* < 0.001) and (to a somewhat lesser extent) with the two indicators of well-being (*r*_s_ range from 0.385 to 0.422, all *p* < 0.001). A similar pattern could be observed for FGA: even slightly stronger correlations with the two indicators of subjective well-being were obtained (self-esteem: *r*_*s*_ = 0.616, satisfaction with life: *r*_*s*_ = 0.649, both *p* < 0.001). The two operationalizations of humor were differentially associated with flexible goal adjustment. As expected, the association for MSHS was slightly lower (*r*_s_ = 0.337, *p* < 0.001) than for HCOP (*r*_s_ = 0.491, *p* < 0.001).

**Table 1:** Means, standard deviations, minimum, maximum, and bivariate correlations of main study variables.

	***M***	***SD***	***Min***	***Max***	**Correlations**	**1**		**2**		**3**		**4**		**5**		**6**	
1. AEUL	—	—	0	1	*r*_s_	—											
					*p*	—											
2. SBUL	3.760	0.769	1.556	5.000	*r*_s_	0.069		—									
					*p*	0.404		—									
3. RSES	3.070	0.746	1.200	4.000	*r*_s_	−0.359	^***^	−0.248	^**^	—							
					*p*	<0.001		0.002		—							
4. SWLS	24.392	6.805	7.000	35.000	*r*_s_	−0.321	^***^	−0.239	^**^	0.646	^***^	—					
					*p*	<0.001		0.003		<0.001		—					
5. MSHS	3.564	0.754	1.706	4.941	*r*_s_	−0.246	^**^	−0.033		0.397	^***^	0.385	^***^	—			
					*p*	0.003		0.687		<0.001		<0.001		—			
6. HCOP	3.855	0.925	1.250	5.000	*r*_s_	−0.205	^*^	−0.207	^*^	0.422	^***^	0.407	^***^	0.666	^***^	—	
					*p*	0.013		0.011		<0.001		<0.001		<0.001		—	
7. FGA	3.295	0.744	1.333	4.733	*r*_s_	−0.271	^***^	−0.368	^***^	0.616	^***^	0.649	^***^	0.337	^***^	0.491	^***^
					*p*	<0.001		<0.001		<0.001		<0.001		<0.001		<0.001	

*AEUL, actual experience of unrequited love (0 = no, 1 = yes); SBUL, subjective burden by unrequited love; RSES, Rosenberg self-esteem scale; SWLS, satisfaction with life scale; MSHS, Multidimensional Sense of Humor Scale; HCOP, Humorous Change of Perspective; FGA, Flexible Goal Adjustment; r_s_, Spearman's rank correlation coefficient*.

* *p < 0.05*,

** *p < 0.01*,

*** *p < 0.001*.

For a more detailed comparison of the subsamples with current vs. past experience of unrequited love, subgroup medians were compared using the Mann-Whitney-*U*-test (assumptions for an independent *t*-test were not met), exact results are presented in Table 2. In summary, the two subgroups differ regarding the main level of all main study variables except subjective burden due to unrequited love. Irrespective of whether participants provided information regarding a current or past unrequited love, they reported similar levels of burden on average. Regarding the indicators of well-being participants referring to an actual experience reported lower levels than participants referring to a past experience. In terms of coping resources, those currently unhappy in love also report less humor (both operationalizations) and less flexible goal adjustment than those with the experience lying in the past.

**Table 2:** Location parameters of the main study variables for the subsamples with actual and past experience of unrequited love compared by Mann-Whitney-U-Test.

**Variable**	**Actual experience**			**Past experience**			***W***	***p***
	***M***	***SD***	***Mdn***	***M***	***SD***	***Mdn***		
SBUL	3.828	0.796	3.944	3.731	0.760	3.778	2,487.5	0.483
RSES	2.614	0.835	2.600	3.263	0.614	3.350	1,252.5	<0.001
SWLS	20.818	7.753	22.500	25.904	5.765	28.000	1,362.5	<0.001
MSHS	3.254	0.856	3.382	3.695	0.670	3.824	1,577.0	0.003
HCOP	3.506	1.100	3.625	4.002	0.802	4.250	1,699.5	0.013
FGA	2.921	0.879	3.167	3.454	0.619	3.533	1,504.0	0.001

*n(actual) = 44, n(past) = 104; SBUL, subjective burden by unrequited love; RSES, Rosenberg self-esteem scale; SWLS, satisfaction with life scale; MSHS, Multidimensional Sense of Humor Scale; HCOP, Humorous Change of Perspective; FGA, Flexible Goal Adjustment*.

### Humor as a Moderator of the Relationship Between the Burden of Unrequited Love and Subjective Well-Being

**Figure 1:**
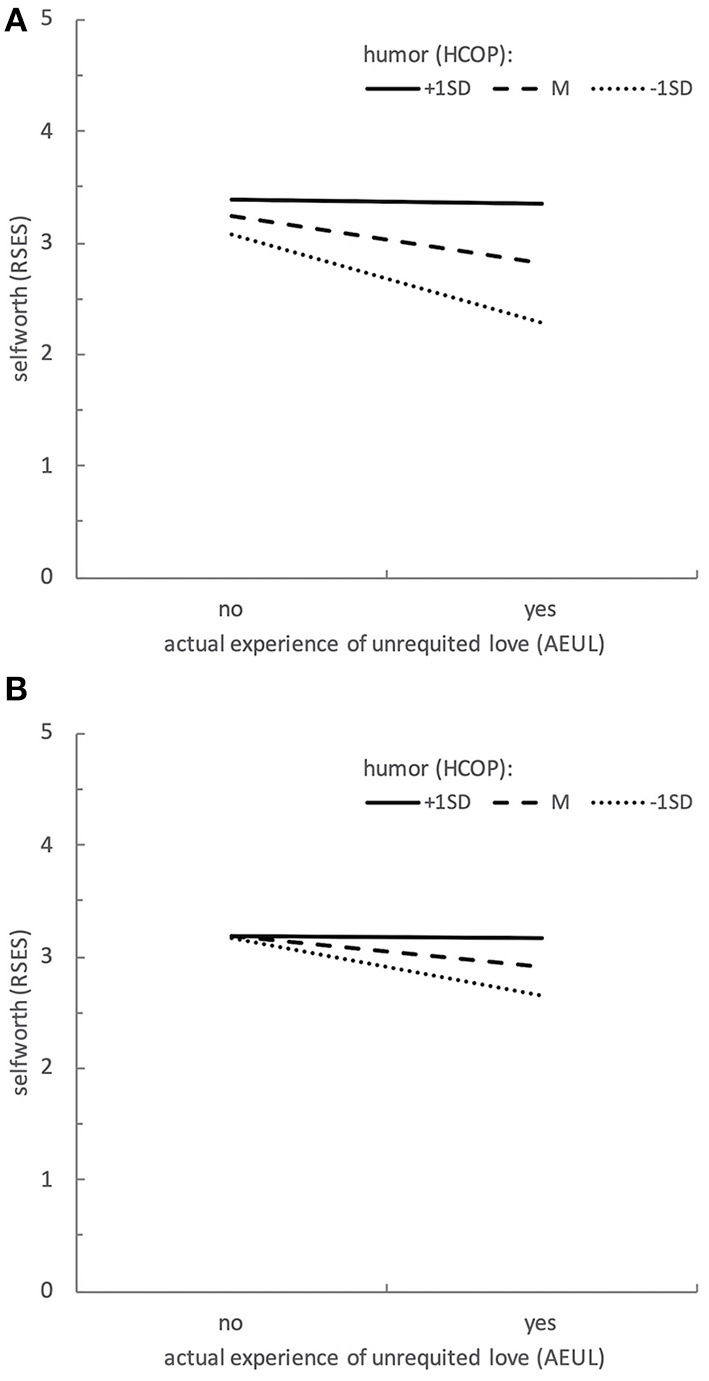
Visualization of moderation effects (Data of Model 3): Predicting Self-esteem (RSES) from actuality of unrequited love (AEUL) and Humorous Change of Perspective (HCOP) **(A)** step 1: no covariate considered in the model **(B)** step 2: Flexible Goal Adjustment (FGA) added as covariate in the model, the figure displays results for FGA fixed to the current sample's mean value.

Detailed results of the multiple regression analyses for self-esteem as an indicator of well-being and criterium in the analyses are presented in Table 3 (MSHS as indicator of humor) and Table 4 (HCOP as indicator of humor). Results for satisfaction with life as indicator of well-being and criterium in the regression analyses parallel these findings and can be found in Supplementary Tables 3, 4. Relevant for the hypothesis 1 regarding humor as a moderator is step 1 of the described hierarchical regression analyses. The main result of these analyses can be summed up as follows: In all but one analysis (Model S4: life satisfaction as criterion, subjective burden by unrequited love, and humorous change of perspective as indicator of humor, $\beta_{\text{burde}{\text{n}}^{*}\text{humor}}$ = 0.081, *p* = 0.274), the interaction of burden^*^humor was a statistically significant predictor of the used indicator of well-being (Models 1–to 4 and S1–S3: $\beta_{\text{burde}{\text{n}}^{*}\text{humor}}$ range from 0.180 to 0.339, all *p* < 0.05). Subsequent simple slope analyses to specify these statistically significant interactions all showed the same pattern (regardless of the indicators used): For expressions of humor one standard deviation above the mean, the association between psychological burden due to unrequited love and the indicator of subjective well-being disappeared; thus, burden was no longer a significant predictor of well-being in this case (β_burden_ range from −0.130 to 0.011, *p* range from 0.226 to 0.945). However, for levels of humor one standard deviation below the mean, burden stayed a statistically significant predictor; thus, higher burden was associated with lower levels of well-being (β_burden_ range from −0.482 to −0.358, all *p* < 0.001). A graphical representation illustrating these moderation-effects for an exemplary combination of indicators (Model 3: actuality of experience of unrequited love, humorous change of perspective, and self-esteem) is depicted in Figure 1A.

**Table 3:** Summary of hierarchical regression analyses predicting self-esteem from burden of unrequited love (two different operationalizations) and sense of humor (measured by MSHS).

	**Variable**	***B***	***SE B***	**β**	***t***	***p***	**95% CI for** ***B***		**Model summaries**
							***LL***	***UL***	
**1. Actuality of unrequited love as predictor (model 1)**									
Step 1	(Intercept)	3.231	0.063		51.356	<0.001	3.107	3.356	*R*^2^ = 0.305 *F*_(3, 144)_ = 21.032 *p* ≤ 0.001
	AEUL	−0.450	0.119	−0.276	−3.786	<0.001	−0.685	−0.215	
	MSHS^a^	0.232	0.093	0.234	2.502	0.013	0.049	0.414	
	AEUL*MSHS^a^	0.317	0.145	0.205	2.178	0.031	0.029	0.604	
Step 2	(Intercept)	1.319	0.229		5.747	<0.001	0.865	1.772	Δ*R*^2^ = 235 *F*_(1, 143)_ = 73.117 *p* ≤ 0.001
	FGA	0.560	0.065	0.558	8.551	<0.001	0.430	0.689	
	AEUL	−0.258	0.100	−0.158	−2.588	0.011	−0.454	−0.061	
	MSHS^a^	0.081	0.078	0.082	1.049	0.296	−0.072	0.235	
	AEUL*MSHS^a^	0.187	0.120	0.121	1.565	0.120	−0.049	0.424	
**2. Subjective burden by unrequited love as predictor (model 2)**									
Step 1	(Intercept)	3.076	0.051		60.714	<0.001	2.976	3.176	*R*^2^ = 0.333 *F*_(3, 144)_ = 23.982 *p* ≤ 0.001
	SBUL^a^	−0.230	0.066	−0.237	−3.473	<0.001	−0.362	−0.099	
	MSHS^a^	0.366	0.069	0.370	5.283	<0.001	0.229	0.503	
	SDUL^a^*MSHS^a^	0.314	0.082	0.270	3.845	<0.001	0.153	0.476	
Step 2	(Intercept)	1.235	0.251		4.920	<0.001	0.739	1.731	Δ*R*^2^ = 0.186 *F*_(1, 143)_ = 55.438 *p* ≤ 0.001
	FGA	0.558	0.075	0.556	7.446	<0.001	0.410	0.706	
	SBUL^a^	−0.037	0.062	−0.038	−0.595	0.553	−0.160	0.086	
	MSHS^a^	0.175	0.064	0.176	2.710	0.008	0.047	0.302	
	SDUL^a^*MSHS^a^	0.138	0.074	0.119	1.879	0.062	−0.007	0.284	

*AEUL, actual experience of unrequited love (0 = no, 1 = yes); SBUL, subjective burden by unrequited love; FGA, Flexible Goal Adjustment; MSHS, Multidimensional Sense of Humor Scale; CI, confidence interval; LL, lower limit; UL, upper limit*.

a *mean-centered*.

**Table 4:** Summary of hierarchical regression analyses predicting self-esteem from burden of unrequited love (two different operationalizations) and Humorous Change of Perspective (measured by HCOP-Scale).

	**Variable**	***B***	***SE B***	**β**	***t***	***p***	**95% CI for** ***B***		**Model summaries**
							***LL***	***UL***	
**1. Actuality of unrequited love as predictor (model 3)**									
Step 1	(Intercept)	3.236	0.058		55.396	<0.001	3.121	3.352	*R*^2^ = 0.398 *F*_(3, 144)_ = 31.683 *p* ≤ 0.001
	AEUL	−0.412	0.110	−0.253	−3.751	<0.001	−0.629	−0.195	
	HCOP^a^	0.172	0.072	0.214	2.411	0.017	0.031	0.314	
	AEUL*HCOP^a^	0.403	0.106	0.339	3.797	<0.001	0.193	0.612	
Step 2	(Intercept)	1.493	0.249		5.993	<0.001	1.000	1.985	Δ*R*^2^ = 0.159 *F*_(1, 143)_ = 51.065 *p* ≤ 0.001
	FGA	0.512	0.072	0.511	7.146	<0.001	0.370	0.654	
	AEUL	−0.266	0.097	−0.163	−2.749	0.007	−0.457	−0.075	
	HCOP^a^	0.005	0.066	0.007	0.081	0.935	−0.125	0.136	
	AEUL*HCOP^a^	0.286	0.093	0.241	3.081	0.002	0.102	0.469	
**2. Subjective burden by unrequited love as predictor (model 4)**									
Step 1	(Intercept)	3.099	0.052		59.553	<0.001	2.997	3.202	*R*^2^ = 0.326 *F*_(3, 144)_ = 23.172 *p* ≤ 0.001
	SBUL^a^	−0.166	0.068	−0.171	−2.436	0.016	−0.301	−0.031	
	HCOP^a^	0.330	0.059	0.410	5.604	<0.001	0.214	0.447	
	SDUL^a^*HCOP^a^	0.199	0.068	0.209	2.925	0.004	0.064	0.333	
Step 2	(Intercept)	1.201	0.261		4.604	<0.001	0.685	1.717	Δ*R*^2^ = 0.186 *F*_(1, 143)_ = 54.522 *p* ≤ 0.001
	FGA	0.573	0.078	0.571	7.384	<0.001	0.419	0.726	
	SBUL^a^	−0.011	0.062	−0.011	−0.170	0.865	−0.133	0.112	
	HCOP^a^	0.111	0.058	0.137	1.894	0.060	−0.005	0.226	
	SDUL^a^*HCOP^a^	0.123	0.059	0.129	2.082	0.039	0.006	0.239	

*AEUL, actual experience of unrequited love (0 = no, 1 = yes); SBUL, subjective burden by unrequited love; FGA, Flexible Goal Adjustment; HCOP, Humorous Change of Perspective; CI, confidence interval; LL, lower limit; UL, upper limit*.

a *mean-centered*.

### Humor as a Possible Coping Resource Beyond Accommodative Flexibility

The effect of adding FGA as a covariate in the multiple regression analyses (addressing hypothesis 2) can be seen in the results of the second step of the hierarchical regression analyses documented in Tables 3, 4 (for self-esteem as criterium) and Supplementary Tables 3, 4 (results for satisfaction with life as criterion). Results differed regarding what indicator of humor was used. For all four models using MSHS as an indicator of humor, the interaction of burden^*^humor was no longer a statistically significant predictor of the respective indicator of well-being (Models 1, 2, S1, S2: $\beta_{\text{burde}{\text{n}}^{*}\text{humor}}$ range from 0.006 to 0.121, *p* range from 0.062 to 0.921). However, for HCOP in all but one analysis (Model S4: the model, that did not provide a statistically significant interaction without controlling for FGA either, $\beta_{\text{burde}{\text{n}}^{*}\text{humor}}$ = −0.010, *p* = 0.871), the interaction of burden^*^humor stayed a statistically significant predictor of the used indicator of well-being (Models 3, 4, S3: $\beta_{\text{burde}{\text{n}}^{*}\text{humor}}$ range from 0.129 to 0.241, all *p* < 0.05). Again, subsequent simple slope analyses were performed to specify these statistically significant interactions. They revealed nearly the same pattern observed without FGA as a covariate. For expressions of humor one standard deviation above the mean, across all three models, slopes did not statistically significant differ from zero, so for this higher level of humor, there was (like in the models without flexible goal adjustment as a covariate), no statistically significant association between the measure of burden and the indicator of well-being used (β_burden_ range from −0.014 to 0.099, *p* range from 0.233 to 0.973). However, for levels of humor one standard deviation below the mean, results differed somewhat. Whilst controlling for FGA, only two out of three slopes showed a statistically significant negative value, demonstrating that for low levels of humor the experienced burden stayed a statistically significant predictor of well-being: a higher burden was associated with lower well-being (Model 3-1SD: β_burden_ = −0.324, *p* < 0.001 and Model S3-1SD: β_burden_ = −0.210, *p* = 0.008). For one indicator-combination (self-esteem as criterion, subjective burden by unrequited love), the slope for levels of humor one standard deviation below the mean did not reach significance (Model 4-1SD: β_burden_ = 0.099, *p* = 0.233). So, in this model, adding flexible goal adjustment into the model lead to a statistically non-significant association of burden and well-being even for a lower level of humor. Again, a graphical representation of the moderation in models that entail flexible goal adjustment as a covariate is depicted in Figure 1B for an exemplary combination of indicators (Model 3: actuality of experience of unrequited love, HCOP, and self-esteem).

## Discussion

The present study had two aims. First, it attempted to replicate the coping effect of humor with respect to a previously unstudied burden: unrequited love as a prototypical example of the experience of blocking of a personally highly important goal. The findings confirm (and replicate) that humor—across two different operationalizations—moderates the relationship between burdens associated with unrequited love and psychological well-being: individuals with higher humor scores had a less pronounced correlation between objective and subjective indicators of this burden and indicators of subjective quality of life, in particular, the sense of self-esteem that plausibly is specifically threatened by unrequited love, but also life satisfaction. These results agree with a number of earlier studies (with respect to a variety of other burdens), demonstrating the buffering effect of humor.

Second, it was examined to which extent this alleviative effect of humor can be attributed to perspective change as a general capacity [i.e., constitutive both for humor and for (other) forms of coping]. For this purpose, we controlled for the effect of a coping resource whose buffering effect is specifically explained by perspective change. With respect to this hypothesis, the findings of the present study depend on the way by which humor was assessed. On the one hand, we found that the burden alleviating effect of the facets addressed by the MSHS (production, social uses, and attitudes toward humor) no longer reached statistical significance once FGA was controlled for. This indicates that at least substantial parts of these facets of humor share similarities with the adaptive processes that are captured by the FGA scale; actually, the bivariate correlation between MSHS and FGA is *r* = 0.337 (see also Thomsen, 2016). It is important to note here that we explicitly excluded the items of the MSHS that is particularly intended to capture the coping effect of humor. This pattern of results seems to suggest that individual use of and a positive attitude toward humor are associated with facets of accommodative coping.

On the other hand, with respect to the Humorous Change of Perspective (HCOP) scale (which we constructed precisely to capture the perspective change facet in humor), we however found that, somewhat contrary to our expectations, the buffering effects of humor on the relationship between the burden of unrequited love and self-esteem or life satisfaction remained largely unchanged after controlling for perspective change (as captured in the FGA scale). Since the bivariate correlation between FGA and HCOP was (as reported above) relatively high (*r* = 0.491), it is likely that HCOP actually has a considerable intersection with FGA. However, the present results seem to indicate that the particular aspect of humorous perspective change captured by the HCOP that contributes to its moderating effect is not fully entailed in the FGA scale. There are several explanations for this pattern of results. Either FGA is effective due to a different facet of its alleviative effect on goal blocking (at least with respect to this particular goal blocking), or change of perspective is not essential for humor (broadly understood), or the change of perspective essential for humor contains another (“own”) facet of change of perspective that is not contained in FGA. It is possible, however, that this pattern of results is caused—at least in part—by the (disputable) validity of the HCOP scale. Several coping humor scale have been published in the literature, some of which also comprise humorous perspective taking (e.g., Martin and Lefcourt, 1983; Ruch et al., 1996; Martin et al., 2003; Ruch and Heintz, 2016); thus, subsequent studies could investigate whether our results can be replicated (or differentiated) utilizing at least some of these scales (or subscales). Given the result that the MSHS coping effect waned once FGA was controlled for, we would expect mixed results for these other scales.

The need for further replication also concerns the (waning of) coping effect for MSHS: Future studies should investigate to what extent this effect can also be shown in relation to other, more differentiated humor scales (Kuiper et al., 2004; Cann and Collette, 2014; Pérez-Aranda et al., 2019; Ruch and Heintz, 2019). As the subscales of the MSHS are highly correlated, this studies used the total score; however, this prevents the investigation of differential effects of subscales. Moreover, all aspects captured in the MSHS relate exclusively to “positive” uses of humor (see Ruch and Heintz, 2016; Heintz et al., 2018; Perchtold et al., 2019), which precludes empirical testing of the hypothesis that the cognitive component of perspective-taking might be effective for coping independently of the (intention of) usage of humor. Third, in the context of such extension and replication studies, it would be important to consider other burdens or threats, especially those that have the character of chronic goal blockages (e.g., involuntary unemployment, chronic illness, etc.).

Finally, to more precisely test the assumptions that the ability to change perspective are important effective factors for both humor and coping, and that some forms of humor additionally show an incremental coping effect, it would be necessary to measure perspective change more directly, as we only measured this assumption indirectly in this study. Studies, which directly test the presumed underlying mental ability of perspective change, have been rare so far. With respect to cognitive (re)appraisal (for which, in turn, perspective change may be a necessary condition, as argued above), the study by Samson et al. (2014) has provided evidence that humor still has an incremental relief effect even when a “sober” re-appraisal is controlled for (see Perchtold et al., 2019 for a similar approach with respect to personality).

The replicability of the role of perspective change with regard to the coping effect of humor supports the suggestion, put forward several times (for a summary Martin, 2008; Ruch, 2008; Martin and Ford, 2018), that cognitive adaptation may be a central process in the coping effects of humor. However, it is important to differentiate this interpretation: If the finding of an incremental value of the HCOP should prove replicable as well, this suggests that there are aspects responsible for the moderating effect of humorous change of perspective that are not entailed in the FGA scale. One plausible interpretation of this pattern of results is that it is not primarily the perspective changing facets of FGA that are effective with respect to unrequited love. For instance, another constitutive aspect of FGA is the downgrading of the threatened goal—which is not explicitly captured in the HCOP items. At the same time, the alleviating effect of HCOP suggests that (humorous) perspective change actually is important in this respect. Of course, there are further plausible candidates which can explain the coping effects of (several forms of) humor. For instance, Martin (2007; see also Martin and Ford, 2018) had named emotional processes, both physiologically (laughter) and psychologically (enjoyment), as possible effective factors in addition to cognitive adaptations (see also Lefcourt et al., 1995).

## Limitations

Several caveats should be noted with respect to the interpretation of the present findings. First, it should be noted that the present data are cross-sectional; although the cross-sectional relationships reported here can be seen as necessary conditions for (claiming) the tested hypotheses, a longitudinal replication of the present study would be particularly important in order to investigate the assumed causal relation. More importantly, future studies should experimentally vary the facets presumed to be responsible for the buffer effect of humor (for example, in intervention studies) so that causal relationships can also be properly tested.

Second, the sample of the present study is highly likely to be self-selective, possibly in two respects. On the one hand it can be assumed that individuals who experienced a past or current unrequited love as currently unburdening (because of their perspective shifting) were less motivated to participate in the study (“Why should I bother talking about my misguided illusion?”). In support of this, we found that participants who were currently unhappily in love had a lower FGA score (which might indicate such a self-selection). This could lead to an underestimation of the alleviative effect of humor through its perspective-changing aspects in the current study's sample. On the other hand, persons who are currently—or still—heavily suffering from an unrequited love may not be inclined to participate in such a study either (“It hurts to much—I'm not willing to talk about it”). This kind of selection could possibly restrict the variance both of the criterion and the moderator, and, as a consequence, could hamper the detection of the predicted patterns. This is another important argument for a longitudinal replication of the results of this study.

Third, it has not been our intention to identify one form of humor that is effective for coping—or more specifically: for coping by a change of perspective; instead, we investigated whether one essential (at least constitutive for several forms of humor) component of humor could be responsible for its general buffering effect, which presumably emerges differently in different forms of humor. This approach presumes, however, that various forms of humor (e.g., malevolent vs. benevolent) are not truly separate competencies or capacities, but rather differently composed versions of a family of basal competencies (e.g., perspective change). We have not, also for reasons of space, discussed this assumption in detail (it was implied rather than explicit in the introductory remarks). However, since this is an untested assumption, it would be particularly important to examine different forms of humor (and the corresponding forms of assessment). With respect to this very point, our decision to replicate the coping effect of humor using the MSHS unidimensionally is attackable, and certainly to be viewed as a first investigatory step. Although this usage has, arguably, a conservative effect with respect to this effect, and although the excellent internal consistency (Cronbach's α = 0.92) supports this usage, the factor analysis (as presented in the Supplementary Materials) underscores the position that humor (as assessed by the 17 “remaining” items of the MSHS) is a heterogeneous concept. This, in turn supports the argument that it is necessary to investigate in more detail (using more differentiated and modern forms of assessment of humor) which components of humor are effective with respect to its coping effect—and which of these might rest, generally or partly, on perspective change.

Fourth, we operationalized the independent variable (burden of unrequited love) in two very different ways. First, we asked about the subjective burden of the event, second, we chose the temporal distance to the event as a (rough) estimator of burden (because empirical data show that burden decreases over time; see section Introduction). Both indicators of burden have limitations. The subjective burden (retrospectively reported for the majority of participants; *n* = 104) might be confounded by the very coping resources examined here (i.e., perspective change). It is thus plausible that perspective change may have already influenced the current and especially the retrospective assessment of the burden of unrequited love. In the present study, the correlation between FGA and subjective burden from unrequited love in the present study is *r* = −0.368. That is why we chose (in a cross-sectional study) temporal distance as a more “objective” indicator of burden. This indicator is also not entirely independent of the process under study (the more effective the available resources, the greater and/or faster the reduction of burden over time), but here, in any case, a direct confounding of the specification itself with the moderators or the dependent variables is not to be expected here. Both weaknesses in the operationalization of the independent variable are methodologically conservative in the sense that they make the interaction (buffer) effect to be tested more difficult to detect because the statistical or causal relationship between predictor and moderator might obscure the separate effect of the moderator and the interaction effect. Note, however, that the retrospective bias for the subjective burden, if it was indeed relevant for this sample, did not impair the buffering effect of humor in this relationship; this underscores the interpretation that it is not the cognitive perspective shifting component of humor alone that produces the alleviative effect of humor.

Fifth, the present study assessed the individual's ability and inclination with respect to coping-relevant perspective shifts exclusively by the Flexible Goal Adjustment questionnaire; it cannot be ruled out that other instruments that assess or entail coping-relevant perspective change might better capture—and thus partial out more effectively—this coping-significant aspect of humor as assessed by the MSHS and HCOP (With the wisdom of hindsight, it might have been more prudent to already include, on the one hand, at least one of these scales in the present study instead of developing a new and untested one. In addition, it might have been beneficial to not exclude items of the MSHS in the assessment). As discussed above, this underscores the importance of a more detailed and comparative investigation of different humor facets and their operationalization if one wants to better understand what underlies the effect of humor as a coping resource.

## Conclusion and Outlook

The present study, despite its limitations, suggests, first, that the coping efficacy of humor does indeed rest, at least in part, on the adaptive (i.e., accommodative) capacities of the individual. At the same time it is worth considering that the alleviative effect of humor might be based less on the mere cognitive forms of perspective shifting, but also on other humor-specific factors that need to be determined. If these results prove to be replicable with respect to other problems or burdens that entail the blocking of important goals as well as other components of humor (beyond perspective shifting), future studies should distinguish which of these processes contribute to the coping-effect of humor. It is thus of particular importance to refer to experimental designs with respect to the burden and to the (usage of) coping processes. In addition, it would be valuable if these processes could be assessed by measures that do not rely entirely on self-report data. Since humor seems to be a useful coping resource in everyday life, this avenue of research is certainly worth pursuing—not only in terms of theoretical knowledge, but also because of the numerous possible applications, especially in the field of preventative strategies for mental health, which aims to strengthen everyday coping resources and thus promote mental well-being. For example, if future studies confirm that a change of perspective is a relevant, possibly constitutive component of both humor and coping, training program with respect to perspective change (at an early age), perhaps in analogy to creativity training, could be a functional preventive approach in several respects, especially when faced with problems and challenges that cannot be solved by strategic action. This, of course, requires not only more specific knowledge about the developmental conditions and supportability of an individual's ability to change perspective, but above all the replication and differentiated examination of the findings presented here.

## Data Availability Statement

The raw data supporting the conclusions of this article will be made available by the authors, without undue reservation.

## Ethics Statement

The studies involving human participants were reviewed and approved by Ethics Committee of the Faculty of Educational and Social Sciences, University of Hildesheim. The patients/participants provided their written informed consent to participate in this study.

## Author Contributions

The present study was planned by JH and WG. The study was conducted by JH. The results were analyzed by FR and JH, the first draft of the method and results sections were written by FR. The first draft of the introduction and the discussion were written by WG. All authors contributed to all parts of the text and agree to be countable on this study and paper.

## Conflict of Interest

The authors declare that the research was conducted in the absence of any commercial or financial relationships that could be construed as a potential conflict of interest.

## Footnotes

^1^We used the MSHS (Thorson and Powell, 1993) and the SHQ-6-R (Svebak, 2010; see also Martin and Ford, 2018) as well as a self-constructed scale. Due to inconsistencies in the implementation of the response format, the SHQ-6-R could not be properly evaluated. However, the results of the other two scales are reported here in full.

## References

[B1] Abel M. H. (2002). Humor, stress, and coping strategies. Humor 15, 365–381. 10.1515/humr.15.4.365

[B2] Aiken L. S. West S. G. (1991). Multiple Regression: Testing and Interpreting Interactions. Newbury Park, CA: Sage.

[B3] Baumeister R. F. Leary M. R. (1995). The need to belong: desire for interpersonal attachments as a fundamental human-motivation. Psychol. Bull. 117, 497–529. 10.1037/0033-2909.117.3.497 7777651

[B4] Baumeister R. F. Wotman S. R. (1992). Breaking Hearts – The Two Sides of Unrequited Love. New York, NY: The Guilford Press.

[B5] Baumeister R. F. Wotman S. R. Stillwell A. M. (1993). Unrequited love: on heartbreak, anger, guilt, scriptlessness, and humiliation. J. Pers. Soc. Psychol. 64, 377–394. 10.1037/0022-3514.64.3.377

[B6] Blackhart G. C. Nelson B. C. Knowles M. L. Baumeister R. F. (2009). Rejection elicits emotional reactions but neither causes immediate distress nor lowers self-esteem: a meta-analytic review of 192 studies on social exclusion. Pers. Soc. Psychol. Rev. 13, 269–309. 10.1177/1088868309346065 19770347

[B7] Brandtstädter J. (2006). “Action perspectives on human development,” in Handbook of Child Psychology, Vol. 1, Theoretical Models of Human Development, ed Lerner R. M. (New York, NY: Wiley), 516–568.

[B8] Brandtstädter J. Renner G. (1990). Tenacious goal pursuit and flexible goal adjustment: explication and age-related analysis of assimilative and accommodative strategies of coping. Psychol. Aging 5, 58–67. 10.1037//0882-7974.5.1.58 2317302

[B9] Brandtstädter J. Rothermund K. (2002). The life-course dynamics of goal pursuit and goal adjustment: a two-process framework. Dev. Rev. 22, 117–150. 10.1006/drev.2001.0539

[B10] Bringle R. G. Winnick T. Rydell R. J. (2013). The prevalence and nature of unrequited love. SAGE Open 3, 1–15. 10.1177/2158244013492160

[B11] Cann A. Collette C. (2014). Sense of humor, stable affect, and psychological well-being. Eur. J. Psychol. 10, 464–479. 10.5964/ejop.v10i3.746

[B12] Cann A. Holt K. Calhoun L. G. (1999). The roles of humor and sense of humor in responses to stressors. Humor. 12, 177–193. 10.1515/humr.1999.12.2.177

[B13] Carroll N. (2014). Humor. A Very Short Introduction. Oxford, UK: Oxford University Press.

[B14] Charlot N. H. Balzarini R. N. Campbell L. J. (2020). The association between romantic rejection and change in ideal standards, ideal flexibility, and self-perceived mate value. Soc. Psychol. 51, 116–126. 10.1027/1864-9335/a000392

[B15] Craik K. H. Lampert M. D. Nelson A. J. (1996). Sense of humor and styles of everyday humorous conduct. Humor. 9, 273–302.

[B16] Diener E. (1984). Subjective well-being. Psychol. Bull. 95, 542–575. 10.1037/0033-2909.95.3.542 6399758

[B17] Diener E. Emmons R. A. Larsen R. J. Griffin S. (1985). The satisfaction with life scale. J. Pers. Assess. 49, 71–75. 10.1207/s15327752jpa4901_13 16367493

[B18] Eisenberger N. I. (2015). Social pain and the brain: controversies, questions, and where to go from here. Annu. Rev. Psychol. 66, 601–629. 10.1146/annurev-psych-010213-115146 25251482

[B19] Felser G. (2007). “Entwicklung in partnerschaften,” in Entwicklungspsychologie der Lebensspanne, ed Brandtstädter J. (Stuttgart: Kohlhammer), 446–482.

[B20] Ferring D. Filipp S.-H. (1996). Messung des Selbstwertgefühls: befunde zu reliabilität,validität und stabilität der rosenberg-skala. Diagnostica 42, 284–292.

[B21] Fritz H. L. Russek L. N. Dillon M. M. (2017). Humor use moderates the relation of stressful life events with psychological distress. Pers. Soc. Psychol. Bull. 43, 845–859. 10.1177/0146167217699583 28903671

[B22] Gerber J. Wheeler L. (2009). On being rejected: a meta-analysis of experimental research on rejection. Perspect. Psychol. Sci. 4, 468–488. 10.1111/j.1745-6924.2009.01158.x 26162220

[B23] Greve W. Leipold B. Kappes C. (2017). Fear of crime in old age: a sample case of resilience? J. Gerontol. B. Psychol. Sci. Soc. Sci. 73, 1224–1232. 10.1093/geronb/gbw169 28044003

[B24] Heckhausen J. Wrosch C. Schulz R. (2010). A motivational theory of life-span development. Psychol. Rev. 117, 32–60. 10.1037/a0017668 20063963 PMC2820305

[B25] Heintz S. Ruch W. (2019). From four to nine styles: an update on individual differences in humor. Pers. Indiv. Differ. 141, 7–12. 10.1016/j.paid.2018.12.008

[B26] Heintz S. Ruch W. Platt T. Pang D. Carretero-Dios H. Dionigi A.. (2018). Psychometric comparisons of benevolent and corrective humor across 22 countries: the virtue gap in humor goes international. Front. Psychol. 9:92. 10.3389/fpsyg.2018.00092 29479326 PMC5812205

[B27] Hurley M. M. Dennett D. C. Adams R. B. (2011). Inside Jokes. Using Humor to Reverse-Engineer the Mind. Cambridge, MA: MIT Press.

[B28] Janke S. Glöckner-Rist A. (2014). Deutsche Version der Satisfaction with Life Scale (SWLS). Zusammenstellung Sozialwissenschaftlicher Items und Skalen (ZIS). 10.6102/zis147

[B29] JASP Team (2020). JASP (Version 0.14.1) (Computer Software). Available online at: https://jasp-stats.org/download/ (accessed December 4, 2020).

[B30] José H. Parreira P. Thorson J. A. Allwardt D. (2007). A factor-analytic study of the multidimensional sense of humor scale with a portuguese sample. North Am. J. Psychol. 9, 595–610. Available online at: https://www.researchgate.net/publication/230766921_A_Factor-Analytic_Study_of_the_Multidimensional_Sense_of_Humor_Scale_with_a_Portuguese_Sample

[B31] Kross E. Berman M. G. Mischel W. Smith E. E. Wager T. D. (2011). Social rejection shares somatosensory representations with physical pain. Proc. Natl. Acad. Sci. U. S. A. 108, 6270–6275. 10.1073/pnas.1102693108 21444827 PMC3076808

[B32] Kuiper N. A. (2012). Humor and resiliency: towards a process model of coping and growth. Eur. J. Psychol. 8, 475–491. 10.5964/ejop.v8i3.464

[B33] Kuiper N. A. Grimshaw M. Leite C. Kirsh G. (2004). Humor is not always the best medicine: specific components of sense of humor and psychological well-being. Humor 17, 135–168. 10.1515/humr.2004.002

[B34] Kuiper N. A. Martin R. A. Olinger L. J. (1993). Coping humor, stress, and cognitive appraisals. Can. J. Behav. Sci. 25, 81–96. 10.1037/h0078791

[B35] Kuiper N. A. McKenzie S. D. Belanger K. A. (1995). Cognitive appraisals and individual differences in sense of humor: motivational and affective implications. Pers. Indiv. Differ. 19, 359–372. 10.1016/0191-8869(95)00072-E

[B36] Lazarus R. S. Folkman S. (1984). Stress, Appraisal, and Coping. New York, NY: Springer.

[B37] Leary M. R. (2015). Emotional responses to interpersonal rejection. Dialog. Clin. Neurosci. 17, 435–441. 10.1093/acprof:oso/9780195130157.003.0006 PMC4734881 26869844

[B38] Lefcourt H. M. Davidson K. Shepherd R. Philipps M. Prkachin K. M. Mills D. E. (1995). Perspective-taking humor: accounting for stress moderation. J. Soc. Clin. Psychol. 14, 373–391. 10.1521/jscp.1995.14.4.373

[B39] Lefcourt H. M. Martin R. A. (1986). Humor and Life Stress: Antidote to Adversity. New York, NY: Springer.

[B40] Leiner D. J. (2020). SoSci Survey (Version 3.2.18) (Computer Software). Available online at: https://www.soscisurvey.de (accessed November 24, 2020).

[B41] Leist A. K. Müller D. (2013). Humor types show different patterns of self-regulation, self- esteem, and well-being. J. Happiness Stud. 14, 551–569. 10.1007/s10902-012-9342-6

[B42] Luciano E. C. Orth U. (2017). Transitions in romantic relationships and development of self-esteem. J. Pers. Soc. Psychol. 112, 307–328. 10.1037/pspp0000109 27379474

[B43] Martin R. A. (2007). The Psychology of Humor. An Integrative Approach. London: Academic Press.

[B44] Martin R. A. (2008). “Humor and health,” in The Primer of Humor Research, ed Raskin V. (Berlin: Mouton de Gruyter), 479–521. 10.1515/9783110198492.479

[B45] Martin R. A. Ford T. (2018). The Psychology of Humor. An Integrative Approach, 2nd Edn. London: Academic Press.

[B46] Martin R. A. Lefcourt H. M. (1983). Sense of humor as a moderator of the relation between stressors and moods. J. Pers. Soc. Psychol. 45, 1313–1324. 10.1037/0022-3514.45.6.1313

[B47] Martin R. A. Puhlik-Doris P. Larsen G. Grey J. Weir K. (2003). Individual differences in uses of humor and their relation to psychological well-being: development of the Humor Styles Questionnaire. J. Res. Pers. 37, 48–75. 10.1016/S0092-6566(02)00534-2

[B48] Nezu A. M. Nezu C. M. Blissett S. E. (1988). Sense of humor as a moderator of the relation between stressful events and psychological distress: a prospective analysis. J. Pers. Soc. Psychol. 54, 520–525. 10.1037/0022-3514.54.3.520 3361423

[B49] Ozyesil Z. (2012). The prediction level of self-esteem on humor style and positive-negative affect. Psychol. 3, 638–641. 10.4236/psych.2012.38098

[B50] Park L. E. Sanchez D. T. Brynildsen K. (2011). Maladaptive responses to relationship dissolution: the role of relationship contingent self-worth. J. Appl. Soc. Psychol. 41, 1749–1773. 10.1111/j.1559-1816.2011.00769.x

[B51] Perchtold C. M. Weiss E. M. Rominger C. Feyaerts K. Ruch W. Fink A.. (2019). Humorous cognitive reappraisal: more benign humour and less “dark” humour is affiliated with more adaptive cognitive reappraisal strategies. PLoS ONE 14:e0211618. 10.1371/journal.pone.0211618 30703148 PMC6355006

[B52] Pérez-Aranda A. Hofmann J. Feliu-Soler A. Ramírez-Maestre C. Andrés-Rodríguez L. Ruch W.. (2019). Laughing away the pain: a narrative review of humour, sense of humour and pain. Eur. J. Pain. 23, 220–233. 10.1002/ejp.1309 30176100

[B53] Rosenberg M. (1965). Society and the Adolescent Self-Image. Princeton, NJ: Princeton University Press.

[B54] Ruch W. (2008). “Psychology of humor,” in The Primer of Humor Research, ed Raskin V. (Berlin: Mouton de Gruyter), 17–100. 10.1515/9783110198492.17

[B55] Ruch W. Heintz S. (2013). Humour styles, personality and psychological well-being: What's humour got to do with it? Eur. J. Humour Res. 1, 1–24. 10.7592/EJHR2013.1.4.ruch

[B56] Ruch W. Heintz S. (2016). The virtue gap in humor: exploring benevolent and corrective humor. Transl. Issues Psychol. Sci. 2, 35–45. 10.1037/tps0000063

[B57] Ruch W. Heintz S. (2019). “Humor production and creativity: overview and recommendations,” in Explorations in Creativity Research. Creativity and Humor, eds Luria S. R. Baer J. Kaufman J. C. (Cambridge, MA: Elsevier Academic Press), 1–42. 10.1016/B978-0-12-813802-1.00001-6

[B58] Ruch W. Heintz S. Platt T. Wagner L. Proyer R. T. (2018a). Broadening humor: comicstyles differentially tap into temperament, character, and ability. Front. Psychol. 9:6. 10.3389/fpsyg.2018.00006 29403416 PMC5778606

[B59] Ruch W. Hofmann J. Rusch S. Stolz H. (2018b). Training the sense of humor with the 7 Humor Habits Program and satisfaction with life. Humor 31, 287–309. 10.1515/humor-2017-0099

[B60] Ruch W. Kohler G. van Thriel C. (1996). Assessing the “humorous temperament”: construction of the facet and standard trait forms of the State-Trait-Cheerfulness-Inventory — STCI. Humor 9, 303–339. 10.1515/humr.1996.9.3-4.303

[B61] Ruch W. Proyer R. Weber M. (2010). Humor as a character strength among the elderly: empirical findings on age-related changes and its contribution to satisfaction with life. Z. Gerontol. Geriatr. 43, 13–18. 10.1007/s00391-009-0090-0 20012063

[B62] Rühs F. Greve W. Kappes C. (2017). Coping with criminal victimization and fear of crime: the protective role of accommodative self-regulation. Legal Criminol. Psychol. 22, 359–377. 10.1111/lcrp.12106

[B63] Samson A. C. Glassco A. L. Lee I. A. Gross J. J. (2014). Humorous coping and serious reappraisal: short-term and longer-term effects. Eur. J. Psychol. 10, 571–581. 10.5964/ejop.v10i3.730

[B64] Sousa L. M. M. Marques-Bieira C. M. A. Severino S. S. P. Pozo-Rosade J. L. Antunes A. V. Jose H. M. G. (2018). Validation of the multidimensional sense of humor scale with chronic kidney disease. J. Nurs. Educ. Pract. 8, 72–79. 10.5430/jnep.v8n3p72

[B65] Strick M. Holland R. W. van Baaren R. B. van Knippenberg A. (2009). Finding comfort in a joke: consolatory effects of humor through cognitive distraction. Emotion 4, 574–578. 10.1037/a0015951 19653782

[B66] Svebak S. (1996). The development of the Sense of Humor Questionnaire: from SHQ to SHQ-6. Humor 9, 341–361. 10.1515/humr.1996.9.3-4.341

[B67] Svebak S. (2010). The Sense of Humor Questionnaire: conceptualization and review of 40 years of findings in empirical research. Eur. J. Psychol. 6, 288–310. 10.5964/ejop.v6i3.218

[B68] Svebak S. Kristoffersen B. Aasaroed K. (2006). Sense of humor and survival among a county cohort of patients with end-stage renal failure: a two-year prospective study. Int. J. Psychiatry Med. 36, 269–281. 10.2190/EFDR-CMDW-X8MH-WKUD 17236695

[B69] Szabo A. (2007). “Comparison of the psychological effects of exercise and humor,” in Mood and Human Performance: Conceptual, Measurement and Applied Issues, ed Andrew M. L. (New York, NY: Nova Science), 201–216.

[B70] Thomsen T. (2016). Flexible goal adjustment from late childhood to late adolescence: developmental differences and relations to cognitive coping and emotion regulation. Int. J. Dev. Sci. 10, 57–72. 10.3233/dev-150167

[B71] Thomsen T. Fritz V. Mößle R. Greve W. (2015). The impact of accommodative coping on well-being in childhood and adolescence: longitudinal findings. Int. J. Behav. Dev. 39, 467–476. 10.1177/0165025414551762

[B72] Thorson J. A. Powell F. C. (1993). Development and validation of a multidimensional sense of humor scale. J. Clin. Psychol. 49, 13–23. 10.1002/1097-4679(199301)49:1<13::aid-jclp2270490103>3.0.co;2-s 8425929

[B73] Thorson J. A. Powell F. C. Sarmany-Schuller I. Hampes W. P. (1997). Psychological health and humor. J. Clin. Psychol. 53, 605–619. 10.1002/(sici)1097-4679(199710)53:6<605::aid-jclp9>3.0.co;2-i 9316815

[B74] Vaillant G. E. (1993). The Wisdom of the Ego. Cambridge, MA: Harvard University Press.

[B75] Waller K. L. MacDonald T. K. (2010). Trait self-esteem moderates the effect of initiator status on emotional and cognitive responses to romantic relationship dissolution. J. Pers. 78, 1271–1299. 10.1111/j.1467-6494.2010.00650.x 20545823

[B76] Williams K. D. Nida S. A. (eds.). (2017). Ostracism, Exclusion, and Rejection. New York, NY: Routledge.

[B77] Wrosch C. Scheier M. F. (2020). “Adaptive self-regulation, subjective well-being, and physical health: the importance of goal adjustment capacities,” in Advances in Motivation, ed Elliot A. J. (Amsterdam: Elsevier), 199–238.

