# Supplementary material for: Humorous Coping With Unrequited Love: Is Perspective Change Important?
Source: Front Psychol. 2021 Jun 25;12:653900. doi: 10.3389/fpsyg.2021.653900 (PMC8267057; doi:10.3389/fpsyg.2021.653900)
Supplement: Supplementary file 1 [file Table_1.pdf]

Supplementary Material

Supplementary Table 1

Results from exploratory factor analysis of the applied items of the Multidimensional Sense of Humor Scale (MSHS)

MSHS item	Factor loading	
	1	2
Factor 1: humor production and social uses		
Meine Freunde halten mich für eine witzige Person.<br>(I'm regarded as something of a wit by my friends.)	**0.769**	0.285
Ich kann Dinge auf eine Art sagen, die die Leute zum Lachen bringt.<br>(I can say things in such a way as to make people laugh.)	**0.824**	0.192
Meine cleveren Sprüche amüsieren andere.<br>(My clever sayings amuse others.)	**0.731**	0.283
Die Leute erwarten bei mir, dass ich amüsante Dinge sage.<br>(People look to me to say amusing things.)	**0.706**	0.125
Ich nutze Humor, um meine Freunde zu unterhalten.<br>(I use humor to entertain my friends.)	**0.749**	0.300
Ich bin überzeugt davon, dass ich andere Menschen zum Lachen bringen kann.<br>(I'm confident that I can make other people laugh.)	**0.751**	0.233
Andere Leute sagen mir, dass ich lustige Dinge sage.<br>(Other people tell me that I say funny things.)	**0.804**	0.095
Manchmal denke ich mir Witze oder lustige Geschichten aus.<br>(Sometimes I think up jokes or funny stories.)	**0.363**	0.049
Ich kann Menschen mit den Dingen die ich sage oft zum Lachen bringen.<br>(I can often crack people up with the things I say.)	**0.885**	0.090
Ich kann eine angespannte Situation entschärfen, indem ich etwas Lustiges sage.<br>(I can ease a tense situation by saying something funny.)	**0.722**	0.308
Mit meinem Humor kann ich eine gewisse Kontrolle über eine Gruppe von Menschen ausüben.<br>(I can have some control over a group of people by my uses of humor.)	**0.680**	0.145
Factor 2: attitudes towards humor and humorous people		
Ich schätze Leute, die für Humor sorgen.<br>(I appreciate those who generate humor.)	**0.409**	**0.551**
Ich mag gute Witze.<br>(I like a good joke.)	0.085	**0.611**
Jemanden als „Komiker“ zu bezeichnen, ist eine echte Beleidigung. (R)<br>(Calling somebody a “comedian” is a real insult.) (R)	0.009	**0.395**
Ich mag keine Witzbolde. (R)<br>(I dislike comics.) (R)	0.284	**0.816**
Menschen, die Witze erzählen, sind Nervensägen. (R)<br>(People who tell jokes are a pain in the neck.) (R)	0.162	**0.824**
Ich fühle mich unwohl, wenn alle Witze reißen. (R)<br>(I'm uncomfortable when everyone is cracking jokes.) (R)	**0.430**	**0.617**

Note. The extraction method was principal axis factoring with an orthogonal (varimax) rotation. Factor loadings above .30 are in bold. Reverse-scored items are denoted with (R).

Supplementary Table 2

Results from exploratory factor analysis of the self-constructed scale measuring humorous change of perspective (HCOP)

HCOP item	Factor loading
Es fällt mir leicht, eine humorvolle Perspektive einzunehmen.<br>(It is easy for me to take a humorous perspective)	**0.918**
Es gelingt mir schnell, eine humorvolle Perspektive einzunehmen.<br>(I quickly succeed in adopting a humorous perspective)	**0.878**
Ich kann in den meisten Situationen auch eine humorvolle Perspektive einnehmen.<br>(I can adopt a humorous perspective in most situations)	**0.854**
Eine humorvolle Perspektive verleiht mir Leichtigkeit im Leben.<br>(A humorous perspective gives me easiness in life)	**0.683**

Note. The extraction method was principal axis factoring. Factor loadings above .30 are in bold.

Supplementary Table 3

Summary of Hierarchical Regression Analyses predicting Satisfaction with Life (SWLS) from burden of unrequited love (two different operationalizations) and sense of humor (measured by MSHS)

	Variable	*B*	*SE B*	*β*	t	*p*	95% CI for *B* *LL*	95% CI for *B* *UL*	Model summaries
1. actuality of unrequited love as predictor (Model S1)									
step 1	(Intercept)	25.596	0.587		43.581	< .001	24.435	26.757	R^2 = 0.271
	AEUL	-3.229	1.109	-0.218	-2.911	0.004	-5.422	-1.037	*F*(3,144) = 17.852
	MSHS[1]	2.277	0.864	0.252	2.635	0.009	0.569	3.984	*p* = < .001
	AEUL*MSHS[1]	2.785	1.357	0.198	2.052	0.042	0.103	5.466	
step 2	(Intercept)	6.637	2.071		3.205	0.002	2.543	10.731	ΔR^2 = 0.278
	FGA	5.548	0.591	0.607	9.393	< .001	4.380	6.715	*F*(1,143) = 73.117
	AEUL	-1.324	0.899	-0.089	-1.473	0.143	-3.100	0.452	*p* = < .001
	MSHS[1]	0.787	0.700	0.087	1.125	0.263	-0.596	2.171	
	AEUL*MSHS[1]	1.502	1.079	0.107	1.392	0.166	-0.631	3.636	
2. subjective burden by unrequited love as predictor (Model S2)									
step 1	(Intercept)	24.427	0.477		51.162	< .001	23.483	25.370	R^2 = 0.288
	SBUL[1]	-2.067	0.625	-0.234	-3.307	0.001	-3.303	-0.832	*F*(3,144) = 19.387
	MSHS[1]	3.511	0.653	0.389	5.377	< .001	2.220	4.802	*p* = < .001
	SBUL[1]*MSHS[1]	1.913	0.770	0.180	2.485	0.014	0.391	3.436	
step 2	(Intercept)	5.105	2.252		2.267	0.025	0.653	9.557	ΔR^2 = 0.247
	FGA	5.851	0.672	0.640	8.708	< .001	4.523	7.179	*F*(1,143) = 75.830
	SBUL[1]	-0.038	0.558	-0.004	-0.068	0.946	-1.141	1.066	*p* = < .001
	MSHS[1]	1.500	0.578	0.166	2.596	0.010	0.358	2.642	
	SBUL[1]*MSHS[1]	0.065	0.660	0.006	0.099	0.921	-1.239	1.370	

Note. AEUL = actual experience of unrequited love (0 = no, 1 = yes); SBUL = subjective burden by unrequited love; FGA = flexible goal adjustment; MSHS = Multidimensional Sense of Humor Scale; CI = confidence interval; LL = lower limit; UL = upper limit.

[1]mean-centred

Supplementary Table 4

Summary of Hierarchical Regression Analyses predicting Satisfaction with Life (SWLS) from burden of unrequited love (two different operationalizations) and humorous change of perspective (measured by HCOP-Scale)

	Variable	*B*	*SE B*	*β*	t	*p*	95% CI for *B* *LL*	95% CI for *B* *UL*	Model summaries
1. actuality of unrequited love as predictor (Model S3)									
step 1	(Intercept)	25.613	0.555		46.164	< .001	24.516	26.709	R^2 = 0.346
	AEUL	-2.982	1.043	-0.201	-2.858	0.005	-5.043	-0.920	$F(3,144)$ = 25.439
	HCOP[1]	1.911	0.679	0.260	2.813	0.006	0.568	3.254	p = < .001
	AEUL * HCOP[1]	3.076	1.007	0.284	3.053	0.003	1.084	5.067	
step 2	(Intercept)	7.593	2.286		3.321	0.001	3.073	12.112	ΔR^2 = 0.204
	FGA	5.294	0.658	0.579	8.047	< .001	3.993	6.594	$F(1,143)$ = 64.758
	AEUL	-1.474	0.888	-0.099	-1.659	0.099	-3.230	0.283	p = < .001
	HCOP[1]	0.184	0.605	0.025	0.304	0.762	-1.012	1.380	
	AEUL * HCOP[1]	1.868	0.852	0.173	2.192	0.030	0.184	3.552	
2. subjective burden by unrequited love as predictor (Model S4)									
step 1	(Intercept)	24.487	0.489		50.076	< .001	23.520	25.454	R^2 = 0.284
	SBUL[1]	-1.424	0.641	-0.161	-2.222	0.028	-2.691	-0.157	$F(3,144)$ = 19.013
	HCOP[1]	3.262	0.554	0.444	5.889	< .001	2.167	4.357	p = < .001
	SBUL[1]* HCOP[1]	0.701	0.638	0.081	1.099	0.274	-0.560	1.962	
step 2	(Intercept)	4.823	2.348		2.054	0.042	0.181	9.465	ΔR^2 = 0.240
	FGA	5.933	0.698	0.649	8.498	< .001	4.553	7.313	$F(1,143)$ = 72.208
	SBUL[1]	0.188	0.558	0.021	0.337	0.737	-0.914	1.290	p = < .001
	HCOP[1]	0.987	0.526	0.134	1.875	0.063	-0.054	2.027	
	SBUL[1]* HCOP[1]	-0.086	0.530	-0.010	-0.163	0.871	-1.134	0.961	

Note. AEUL = actual experience of unrequited love (0 = no, 1 = yes); SBUL = subjective burden by unrequited love; FGA = flexible goal adjustment; HCOP = humorous change of perspective; CI = confidence interval; LL = lower limit; UL = upper limit.
[1]mean-centred
